# Cardiovascular risk factors and major depressive disorder: a cross-sectional study in São Paulo, Brazil

**DOI:** 10.1590/1516-3180.2020.0054.R1.1802021

**Published:** 2021-06-11

**Authors:** Danielle Bivanco-Lima, Itamar de Souza Santos, Yuan-Pang Wang, Maria Carmen Viana, Laura Helena Andrade, Paulo Andrade Lotufo, Isabela Judith Martins Benseñor

**Affiliations:** I MD, PhD. Professor, Department of Public Health, Faculdade de Ciências Médicas da Santa Casa de São Paulo (FCMSP), São Paulo (SP), Brazil.; II MD, PhD. Center for Clinical and Epidemiological Research, Hospital Universitário (HU), Universidade de Sao Paulo, Sao Paulo, SP, BR, and Professor, Department of Internal Medicine, Faculdade de Medicina FMUSP, Universidade de Sao Paulo, Sao Paulo, SP, BR; III MD, PhD. Assistant Professor, Section of Psychiatric Epidemiology (LIM-23), Institute of Psychiatry, Hospital das Clínicas FMUSP, Faculdade de Medicina, Universidade de São Paulo, Sao Paulo, SP, BR.; IV MD, PhD. Professor, Postgraduate Program on Collective Health, Universidade Federal do Espírito Santo (UFES), Vitória (ES), Brazil.; V MD, PhD. Assistant Professor, Section of Psychiatric Epidemiology (LIM-23), Institute of Psychiatry, Hospital das Clínicas FMUSP, Faculdade de Medicina, Universidade de São Paulo, Sao Paulo, SP, BR.; VI MD, DrPH. Coordinator, Center for Clinical and Epidemiological Research, Hospital Universitário (HU), Universidade de Sao Paulo, Sao Paulo, SP, BR, and Full Professor, Department of Internal Medicine, Faculdade de Medicina FMUSP, Universidade de Sao Paulo, Sao Paulo, SP, BR; VII MD, PhD. Deputy Coordinator, Center for Clinical and Epidemiological Research, Hospital Universitário (HU), Universidade de Sao Paulo, Sao Paulo, SP, BR, and Full Professor, Department of Internal Medicine, Faculdade de Medicina FMUSP, Universidade de Sao Paulo, Sao Paulo, SP, BR.

**Keywords:** Depression, Risk factors, Cardiovascular diseases, Depressive disorder, major, Depressive disorders, Cardiovascular risk, Major depressive disorder, Depressed individuals, Lifestyle and risk factors

## Abstract

**BACKGROUND:**

Cardiovascular risk factors can mediate the association between depression and cardiovascular diseases.

**OBJECTIVE:**

To evaluate cardiovascular risk factors in adult individuals with and without histories of major depression in the metropolitan region of São Paulo, Brazil.

**DESIGN AND SETTING:**

Cross-sectional study in São Paulo (SP), Brazil.

**METHODS:**

This study evaluated 423 individuals without any lifetime diagnosis of major depression and 203 individuals with a previous diagnosis of major depression (n = 626). The participants underwent a psychiatric evaluation using a structured clinical interview (SCID-1), an anthropometric evaluation and a clinical evaluation that included blood pressure measurement and assessment of fasting blood glucose, lipid profile and physical activity levels.

**RESULTS:**

Individuals with histories of major depression were more likely to be female (P < 0.0001). Individuals with lifetime diagnoses of major depression were more likely to be current smokers (odds ratio, OR 1.61; 95% confidence interval, CI 1.01-2.59) and to have diabetes (OR 1.79; 95% CI 1.01-3.21); and less likely to be obese (OR 0.58; 95% CI 0.35-0.94).

**CONCLUSION:**

Individuals with major depression had higher odds of presenting tobacco smoking and diabetes, and lower odds of being obese. Healthcare professionals need to be aware of this, so as to increase the rates of diagnosis and treatment in this population.

## INTRODUCTION

Cardiovascular diseases are the leading cause of death worldwide and in Brazil.^1^ Mortality rates relating to cardiovascular diseases have also been found to be higher in a psychiatric population than in an age-matched general population, with a hazard ratio (HR) of 4.16 (95% confidence interval, CI 1.22-14.25).^2^ There is reasonable data describing the association between depressive disorders and cardiovascular disease incidence and mortality, especially with regard to coronary heart diseases.^3,4^ Nicholson et al. reviewed 54 observational studies and conducted a meta-analysis regarding depression as an etiological factor among individuals with coronary heart diseases. They found that depression increased the risk of incident coronary heart disease (pooled relative risk, RR 1.81; 95% CI 1.53-2.15).^5^

Individuals’ profile of cardiovascular risk factors can explain, at least partially, the higher incidence of coronary heart disease and related mortality among individuals with depressive disorders. Appleton et al. evaluated 8,138 men and found higher incidence of cardiovascular diseases in individuals with depressive disorders (HR 2.36; 95% CI 1.39-4.0). However, the association lost significance after cardiovascular risk factors were included in the model for adjustment (HR 1.73; 95% CI 0.99-2.99).^2^ Therefore, cardiovascular risk factors might be important mediators of the causal model linking depressive disorders and cardiovascular mortality.

Depressed individuals might have unhealthier lifestyles than the general population, with higher rates of smoking and greater physical inactivity.^6,7^ Strine et al. evaluated 217,379 individuals in a survey in the United States and found that individuals with previous major depression had higher odds of current smoking (odds ratio, OR 1.9; 95% CI 1.8-2.1) and physical inactivity (OR 1.3; 95% CI 1.2-1.4).^7^ There are also data showing higher prevalence of metabolic syndrome in patients with major depression^8^ and, more consistently, in depressed women.^9,10^

Studies that have evaluated the relationship between cardiovascular risk factors and depressive disorders in the general population are less common, especially in low and middle-income countries.

## OBJECTIVE

The aim of this study was to evaluate cardiovascular risk factors (hypertension, diabetes, dyslipidemia, smoking, obesity and metabolic syndrome) and their association with lifetime major depression in a subsample of individuals from the São Paulo Megacity Mental Health Survey, in the metropolitan area of São Paulo, Brazil.

## METHODS

### Study design

The São Paulo Megacity Mental Health Survey is a population-based cross-sectional study with the aim of evaluating the prevalence of psychiatric morbidity. It focuses on non-institutionalized Portuguese-speaking adults aged 18 or older, living in the metropolitan area of São Paulo, state of São Paulo, Brazil, and it forms the Brazilian component of the World Mental Health Survey (WMH). This is an initiative from the World Health Organization (WHO) that comprises a series of population-based epidemiological studies of adult resident populations in over 30 participating countries around the world. It is conducted with the same methodological framework, using the same instruments as in the WMH. The study protocol has been detailed elsewhere.^11^

### Study sample

**Figure 1.**
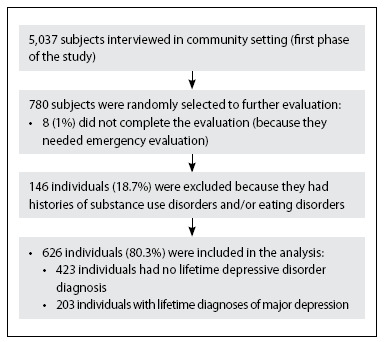
Subjects included in the second phase of the São Paulo Megacity Survey.

The current study was based on a subsample of the Brazilian WMH, with a cross-sectional design and was conducted in parallel with the main household survey. Out of the 5,037 individuals who were evaluated in their households, a random subsample of 780 individuals was selected. Eight individuals (1%) with symptoms that needed emergency evaluation, who did not complete the protocol, were excluded. We also excluded 146 (18.7%) who had substance use disorders or eating disorders and, thus, 626 individuals (80.3%) remained included in the final analysis (**Figure 1**). The only missing data consisted of body mass index (BMI) values for individuals. The remainder of the data was 100% complete.

### Psychiatric and clinical assessments

In the hospital outpatient setting, trained psychiatrists assessed participants through the Structured Clinical Interview for DSM-IV Axis I Disorders (SCID-I).^12^ The SCID-I makes it possible to generate current and lifetime diagnoses on psychiatric conditions. For this analysis, we only included individuals with lifetime diagnoses of major depressive disorder (MDD), with or without associated anxiety disorders.

In addition, the onsite evaluation included a questionnaire that was used to collect the following sociodemographic data: age, sex, marital status, number of years of formal education (measured as number of school years), current job status (worker, housekeeper, student, retired or other) and annual household income. A clinical evaluation focusing on cardiovascular risk factors and cardiovascular diseases was performed by two physicians. All subjects were asked about smoking status (never, past or current), alcohol intake and personal histories of hypertension, diabetes, coronary heart disease or cerebrovascular disease. The level of physical activity was measured using the long version of the International Physical Activity Questionnaire (IPAQ).^13^ Inadequate physical activity was defined as being sedentary or insufficiently active according to the IPAQ, considering two domains separately: leisure-time and journey-to-work (commuting) physical activity.

### Definition of cardiovascular risk factors

Hypertension was defined as mean systolic blood pressure ≥ 140 mmHg, mean diastolic blood pressure ≥ 90 mmHg, medical history of hypertension and/or current use of medication to treat hypertension.^14^ Diabetes was defined as fasting blood glucose ≥ 126 mg/dl, medical history of diabetes and/or use of medication to treat diabetes.^15^

Individuals were considered to have dyslipidemia if they had one or more lipid profile alterations: high-density lipoprotein-cholesterol (HDL-c) levels lower than 40 mg/dl for men and lower than 50 mg/dl for women; triglyceride (TG) levels higher than 150 mg/dl; or low-density lipoprotein-cholesterol (LDL-c) levels higher than 130 mg/dl. Metabolic syndrome was defined as the presence of three or more of the following criteria: abdominal obesity (waist circumference ≥ 102 centimeters (cm) in men and ≥ 88 cm in women); TG blood level ≥ 150 mg/dl; HDL-c cholesterol blood levels lower than 40 mg/dl in men or lower than 50 mg/dl in women; blood pressure (BP) ≥ 130 mmHg of systolic blood pressure and/or ≥ 85 mmHg of diastolic blood pressure; or fasting glucose blood level ≥ 100 mg/dl.^16^

### Anthropometric and blood pressure measurements

The anthropometric evaluation (weight, height and waist circumference) was performed using standardized equipment and techniques.^17^ Blood pressure was measured three times using a standardized technique in the seated position after a five-minute rest. Body mass index (BMI) was calculated as weight in kilograms divided by height in meters squared.^18^ Overweight was defined as BMI between 25 and 29.9 kg/m^2^. Obesity was defined as BMI greater than or equal to 30 kg/m^2^.

### Laboratory measurements

Venous blood samples were obtained after a 12-hour overnight fast. The blood samples were analyzed for the following: glucose levels (hexokinase method); total cholesterol (TC) (enzymatic colorimetric assay); HDL-c (HDL - homogeneous cholesterol); triglycerides (enzymatic colorimetric assay); high-sensitivity C-reactive protein (nephelometry); thyroid-stimulating hormone (TSH); and free thyroxine (immunoenzymatic assay - third generation). LDL-c values were obtained using the Friedewald formula.^16^

### Statistical analysis

Data entry was carried out twice and a validity check was done in order to identify and correct any data entry errors. Groups were compared using the chi-square test or Fisher’s exact test for categorical data, whenever applicable. Continuous variables were tested for normality, and one-way ANOVA (analysis of variance) or nonparametric tests were performed, whenever applicable. We used logistic regression to evaluate comparisons among individuals with and without depression and the results were presented crudely and adjusted for age and sex. The significance level was set at 0.05. We used the SPSS for Windows software, version 16.0 (SPSS Inc., Chicago, United States), and the R software, version 3.0.0 (Vienna, Austria) for the analyses.

### Ethical considerations

The Institutional Review Board of Hospital das Clínicas, Universidade de São Paulo approved the study under the protocol number 234/03 (on April 24, 2003), and all the participants signed a written informed consent statement.

## RESULTS

Among the 626 participants, 383 (61.2%) were female, 423 had no lifetime history of MDD (67.6%; 95% CI 63.8-71.2) and 203 had lifetime histories of MDD (32.4%; 95% CI 28.9-36.2). The mean age was 40.5 years among the individuals without any lifetime diagnosis of major depressive disorder and 41.5 years among the individuals with lifetime histories of major depressive disorder (P = 0.24). A total of 423 individuals (67.6%) had no lifetime history of depressive disorder, while 203 (32.4%) had a SCID-I diagnosis of lifetime depressive disorder. Individuals with lifetime depression were more likely to be current smokers than were individuals who had never been depressed, with borderline significance (20.3% versus 13.0%; P = 0.056) (**Table 1**).

**Table 1:** Cardiovascular disease risk factors according to the presence of lifetime histories of major depressive disorder (MDD)

	Lifetime history of major depressive disorder				P
	No (n = 423)		Yes (n = 203)		
	N	% (95% CI)	N	% (95% CI)	
**Sex**					< 0.001
Female	228	53.9 (49.1-58.6)	155	76.4 (70.1-81.8)	
Male	195	46.1 (41.4-50.9)	48	23.6 (18.2-29.9)	
**Mean age in years (SD)**	40.5 (11.1)	40.5 (39.4-41.6)	41.5 (10.6)	41.5 (40.0-43.0)	0.243
**Smoking status**					0.056
Never	257	60.8 (56.0-65.3)	116	57.1 (50.3-63.8)	
Past	110	26.0 (22.0-30.4)	45	22.2 (16.9-28.3)	
Current	55	13.0 (10.1-16.5)	41	20.2 (15.1-26.1)	
**Body mass index**					0.118
Normal < 25.0 kg/m^2^	149	35.2 (30.8-39.9)	78	38.4 (31.9-45.3)	
Overweight: 25.0-29.9 kg/m^2^	164	38.8 (34.2-43.5)	86	42.4 (35.7-49.3)	
Obese ≥ 30.0 kg/m^2^	107	25.3 (21.3-29.6)	36	17.7 (12.9-23.5)	
**Hypertension**					0.990
No	265	62.6 (58.0-67.2)	128	63.1 (56.3-69.5)	
Yes	158	37.4 (32.8-42.0)	75	36.9 (30.5-43.8)	
**Diabetes mellitus**					0.105
No	390	92.9 (89.3-94.5)	179	88.2 (83.2-92.1)	
Yes	33	7.8 (5.5-10.7)	24	11.8 (7.9-16.8)	
**Dyslipidemia**					0.098
No	159	37.6 (33.1-42.3)	91	44.8 (38.1-51.7)	
Yes	264	62.4 (57.7-66.9)	112	55.2 (48.3-61.9)	
**Metabolic syndrome**					0.788
No	276	65.2 (60.6-69.7)	135	66.5 (59.8-72.7)	
Yes	147	34.8 (30.3-39.4)	68	33.5 (27.3-40.2)	
**Commuting-related physical activity**					0.058
Inactive/sedentary	179	42.3 (37.7-47.1)	105	51.7 (44.9-58.6)	
Insufficiently active	131	31.0 (26.7-35.5)	58	28.6 (22.7-35.1)	
Active	113	26.7 (22.7-31.1)	40	19.7 (11.7-25.6)	
**Leisure-time physical activity**					0.140
Inactive/sedentary	292	69.0 (64.5-73.3)	155	76.4 (70.1-81.8)	
Insufficiently active	56	13.2 (10.3-16.7)	18	8.9 (5.5-13.4)	
Active	75	17.7 (14.3-21.6)	30	14.8 (10.4-20.2)	

95% CI = 95% confidence interval.

There were no differences between the individuals with and without lifetime diagnoses of major depressive disorder regarding cardiovascular risk factors, except for a borderline difference for current tobacco smoking (13.0% among individuals without MDD and 20.2% among individuals with MDD; P = 0.056*)*. Most of the individuals were obese or overweight (64.5% of the individuals without MDD and 61.0% of the individuals with MDD; P = 0.12). High frequencies of hypertension (37% in both groups; P = 0.99), metabolic syndrome (34.8% versus 33.5% in individuals without and with MDD, respectively; P = 0.79) and dyslipidemia (62.4% of individuals without MDD and 55.2% of individuals with MDD; P = 0.10) were observed. There was high frequency of sedentary behavior, particularly with regard to physical activity during leisure time (**Table 1**).

In the logistic regression, crude analysis showed that individuals with lifetime histories of MDD had odds ratios for body mass index, hypertension, diabetes mellitus, dyslipidemia, metabolic syndrome and leisure-time physical activity that were similar to those of individuals without MDD. Also in the crude analysis, individuals with MDD had higher odds ratios for current smoking (OR 1.65; 95% CI 1.04-2.62) and lower odds of active commuting-related physical activity (OR 0.60; 95% CI 0.39-0.93) (**Table 2**).

**Table 2:** Logistic regression models of the association between cardiovascular risk factors and lifetime history of major depression (crude and age and sex-adjusted)

	Presence of lifetime history of major depressive disorder - OR (95% CI)	
	Crude	Age and sex-adjusted
**Body mass index**		
Normal	1.0 (Reference)	1.0 (Reference)
Overweight	1.00 (0.69-1.46)	1.03 (0.69-1.53)
Obese	0.64 (0.40-1.02)	0.58 (0.35-0.94)
**Hypertension**		
No	1.0 (Reference)	1.0 (Reference)
Yes	0.99 (0.70-1.40)	0.98 (0.67-1.44)
**Diabetes mellitus**		
No	1.0 (Reference)	1.0 (Reference)
Yes	1.58 (0.91-2.76)	1.79 (1.01-3.21)
**Dyslipidemia**		
No	1.0 (Reference)	1.0 (Reference)
Yes	0.74 (0.53-1.04)	0.77 (0.54-1.12)
**Metabolic syndrome**		
No	1.0 (Reference)	1.0 (Reference)
Yes	0.95 (0.66-1.35)	0.87 (0.59-1.27)
**Smoking status**		
Never	1.0 (Reference)	1.0 (Reference)
Past	0.91 (0.60-1.37)	0.88 (0.58-1.36)
Current	1.65 (1.04-2.62)	1.61 (1.01-2.59)
**Leisure-time physical activity**		
Inactive/sedentary	(Reference)	(Reference)
Insufficiently active	0.61 (0.34-1.07)	0.73 (0.41-1.30)
Active	0.75 (0.47-1.20)	0.79 (0.49-1.28)
**Commuting-related physical activity**		
Inactive/sedentary	(Reference)	1.0 (Reference)
Insufficiently active	0.75 (0.51-1.12)	0.80 (0.54-1.20)
Active	0.60 (0.39-0.93)	0.66 (0.42-1.03)

OR = odds ratio; 95% CI = 95% confidence interval.

In the age and sex-adjusted logistic regression model, individuals with lifetime major depression had higher odds of being current smokers (OR 1.61; 95% CI 1.01-2.59) and higher odds of presenting diabetes (OR 1.79; 95% CI 1.01-3.21). In addition, individuals with lifetime major depression had lower odds of presenting obesity (OR 0.58; 95% CI 0.35-0.94). No differences were found for other cardiovascular risk factors (hypertension, dyslipidemia, metabolic syndrome and physical activity) (**Table 2**). Further adjustment for cardiovascular risk factors did not change the results (data not shown).

## DISCUSSION

In our study, we found that current smoking and diabetes showed associations with lifetime histories of major depression. We also found lower odds of being obese among individuals with lifetime histories of major depression, compared with nondepressed individuals.

Our study found that individuals with lifetime major depression had higher odds of being diabetic. Most studies have found this same association between diabetes and major depression, including evidence for a bidirectional association.^19,20,21,22,23^ Mommersteeg et al. found among 231,797 individuals from 47 countries that diabetes increased the odds of depressive symptoms (OR 2.36; 95% CI 1.91-2.92).^24^ Mezuk et al. found that diabetes could enhance the risk of incident depression (OR 1.15; 95% CI 1.02-1.30). They also found higher risk of new-onset diabetes among depressed subjects (RR 1.60; 95% CI 1.37-1.88).^23^

There are multiple explanations for the association between diabetes and depression, as mediated by long-term use of antide-pressants^25^ and increased inflammation and insulin resistance.^26^ The recent discussion on syndemics has indicated that the association between diabetes and depression is more complex than it would seem through explanations that invoke exclusive biological pathways. The dynamics of poverty and sociocultural contexts influence lifestyle and the health and disease process that links these two diseases.^27,28,29,30^ Because the present study was a cross-sectional analysis, it is impossible to draw any conclusions relating to causality. Nevertheless, our data confirm the presence of this association in a Brazilian population sample.

We also found higher odds of current tobacco use among individuals with lifetimes of depressive disorder. Other studies have reported similar results.^7,31^ Strine et al. found higher odds of tobacco use among currently depressed subjects (OR 2.2; 95% CI 2.0-2.3) and previously depressed subjects (OR 1.9; 95% CI 1.8-2.1).^7^ Goodwin et al. found that among smokers the prevalence of past-year depression was 10.45%, compared with 5.51% among never-smokers.^32^ Previous Brazilian studies confirmed the association between tobacco smoking and depressive symptoms.^33,34^ Borçói et al. found a prevalence ratio of 2.69 (95% CI 1.62-4.46) for being a current smoker, among individuals with depressive disorder in a sample at a primary care service in a Brazilian municipality (Alegre, Espírito Santo).^33^ Rocha et al. evaluated 1,054 individuals in a population-based survey in a southern municipality (Florianópolis, Santa Catarina) and found that 29% of current smokers had depressive symptoms.^34^

We found lower odds of obesity among individuals with lifetime histories of major depression. The results in the medical literature regarding this association are divergent, with positive results,^35,36^ negative results^37,38,39^ and inverse associations.^40^ A systematic review by Luppino et al. including 15 studies and 58,745 individuals found higher odds of major depression among individuals with obesity (OR 1.57; 95% CI 1.23-2.01) and also reported that individuals with major depression had higher odds of obesity (OR 1.40; 95% CI 1.15-1.71), although no association with overweight was found (OR 0.98; 95% CI 0.83-1.16).^35^ However, Luppino et al. pointed out that differences in study populations might explain the heterogeneous results and suggested that some American studies had a positive association, whereas European studies did not show any significant associations between depression and obesity.^35^ An Asian study also reported an association of depression with underweight among men.^40^ A Brazilian cohort in Pelotas (Rio Grande do Sul) found that individuals with major depressive disorder had higher odds of obesity (OR 1.9; 95% CI 1.09-3.46).^41^ The Brazilian National Health Survey (Pesquisa Nacional de Saúde, PNS 2013) also found a bidirectional association between depression and obesity.^42^ Paulitsch et al. found that the association of depression and obesity was mediated by weight perception, i.e. believing oneself to be fat. They also found that some groups were more vulnerable: being non-single, having more schooling and not engaging in physical activity.^43^

Our data contrasted with different Brazilian studies showing regional heterogeneity. In our study, the association between higher rates of commuting-related physical activity and lifetime history of MDD lost its significance after adjustment. Because our sample had a higher number of women than of men and commuting physical activity occurs more frequently among men than among women,^44,45^ our results probably lost significance after adjustment for sex.

Our study had some methodological strengthens. Trained psychiatrists made the psychiatric diagnoses of all participants, using a gold-standard instrument (SCID-I). The cardiovascular risk factors were evaluated using medical history and using clinical and laboratory assessments from onsite evaluation made by trained physicians.

The results from the study highlight the importance of diagnosing and treating depression within primary and secondary care.^46^ Depression is an important risk factor for noncompliance with treatment of chronic diseases. Therefore, for effective control of cardiovascular risk factors, it is mandatory to recognize and treat depression. Depressive individuals are less active, with higher frequency of smoking and less adherence to prescribed medication.^47^ In addition, practicing physical activity can also be part of depression treatment. On the other hand, the effect of treating depression can have some impact regarding weight gain, which has been shown to have an important relationship with depression and cardiovascular risk factors and chronic diseases. Healthcare professionals can manage treatment of depression using better cardiovascular-profile drugs or considering other treatment options, such as psychotherapy, whenever possible.^48^.

Our study also had some limitations, given that the cross-sectional study design did not permit evaluation of causality. Moreover, the diagnosis of diabetes was made based on only one measurement of fasting plasma glucose, as a consequence of budget limitations and logistic limitations on performing some laboratory tests such as hemoglobin A1c. Another important point is that our sample was young, with a mean age of around 40 years. The prevalence of cardiovascular risk factors increases with age, which may explain the lower frequency in our sample. In addition, although the sample in the first phase of the study with home interviews was representative of the population living in the metropolitan region of São Paulo, only a subsample of participants was invited in the second phase, and many of them refused to participate, thus introducing some selection bias. However, even considering the young age, we reported that there was higher prevalence of diabetes and smoking among the depressive participants than among the nondepressed participants.

## CONCLUSIONS

In this sample of individuals living in the metropolitan region of São Paulo, the biggest city in Brazil, we found that individuals with lifetime major depression had higher odds of current tobacco use and of presenting diabetes mellitus. They also had lower odds of being obese, compared with nondepressed individuals. Healthcare professionals need to increase their awareness of the diagnosis of cardiovascular risk factors among individuals with major depression, in order to enhance preventive strategies and thus reduce morbidity and mortality in this high-risk group.
